# Supplementary material for: Chromothripsis during telomere crisis is independent of NHEJ, and consistent with a replicative origin
Source: Genome Res. 2019 May;29(5):737–49. doi: 10.1101/gr.240705.118 (PMC6499312; doi:10.1101/gr.240705.118)
Supplement: Supplemental Material [file supp_gr.240705.118_Supplemental_file_1.zip › contigs/annotated_contigs/DB82/contig.2.DB82_length_510_mean_cov_5.57843137255.docx]

**DB82_length_510_mean_cov_5.57843137255**

```
AATGAGACAGAGAGGAGGAGGTACAAGGCAGGAGCCACGGCAGAGAGAGGCATGTGGAGTGAGCTTGCATCCAGTGCAGAGGCGTGGGA
 >chr10:43484427-43484783 - E=9e-203 p=2e-02
GGGTGCAGGGCAGACGACATGATCAGCGGGGGCGGACGCCACCACAGCCACTTCCCAGTGTGTTGGAGGCTCTGTTCCTGTTAGGCACT

GTGCATTTCTGAGGGGCTGGCCTGTGACGTGGGGTCTGTGGTTTGGACTCTCTGACCCTGTTCTGCTGCTATGCATAAAGTCAGGGCTG

TAGGGGCCTTAGAGGCCAAGGCCAGAGCTCATTTCACAAAGGCAGCCATGGTGGCCAGAGAGGAAGGGGGTTCTGGACCCCCTAGATGC

|CTCGGCATCAGAGGGAGAGGGAGAGGGAGAGGGAGAGGGAGGGGGAGGGAGAGGGAGAGGGAGAGGGAGAGGGAGAGGGAGAGGGAGA
  >chr3:186581280-186581379 - E=6e-36
GGGAGAGGG|GGCGGGCGCGTCTGTTTCGTCTAGGAGATGCTGGGTGTTTCCGTTTCTGTTTGTTTC
```
